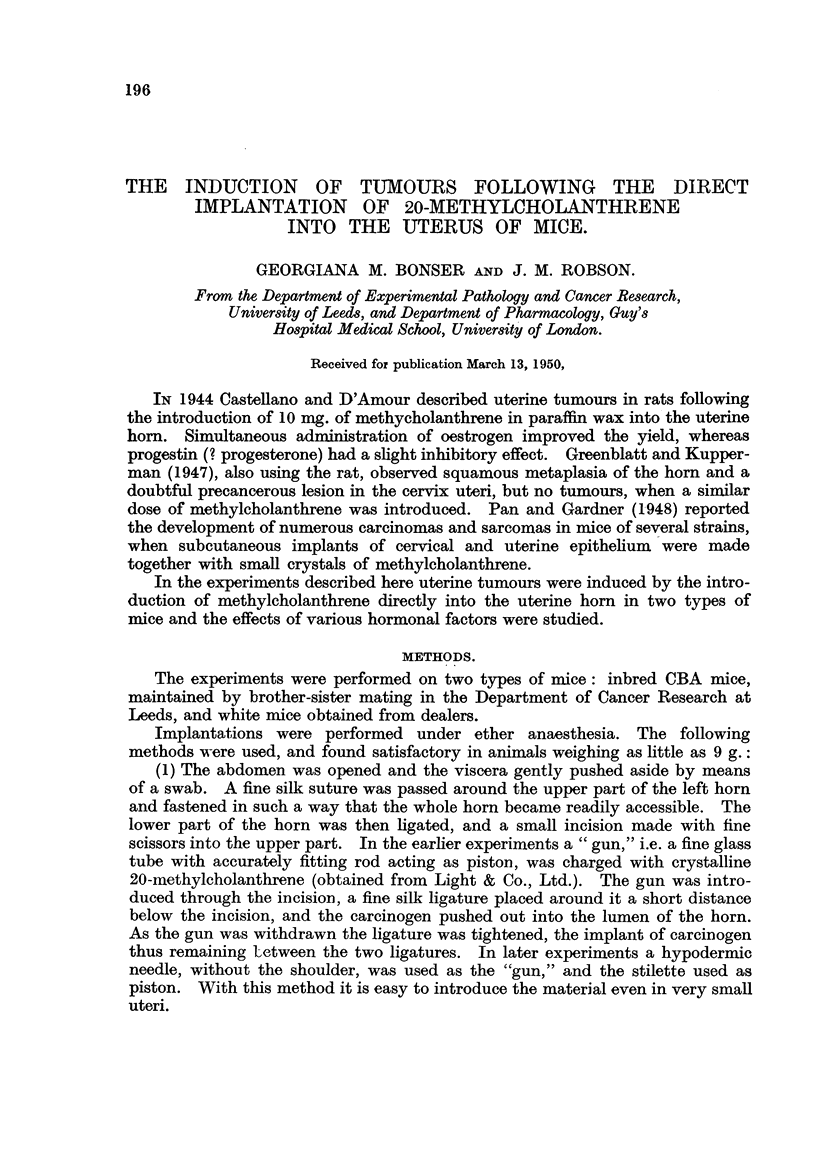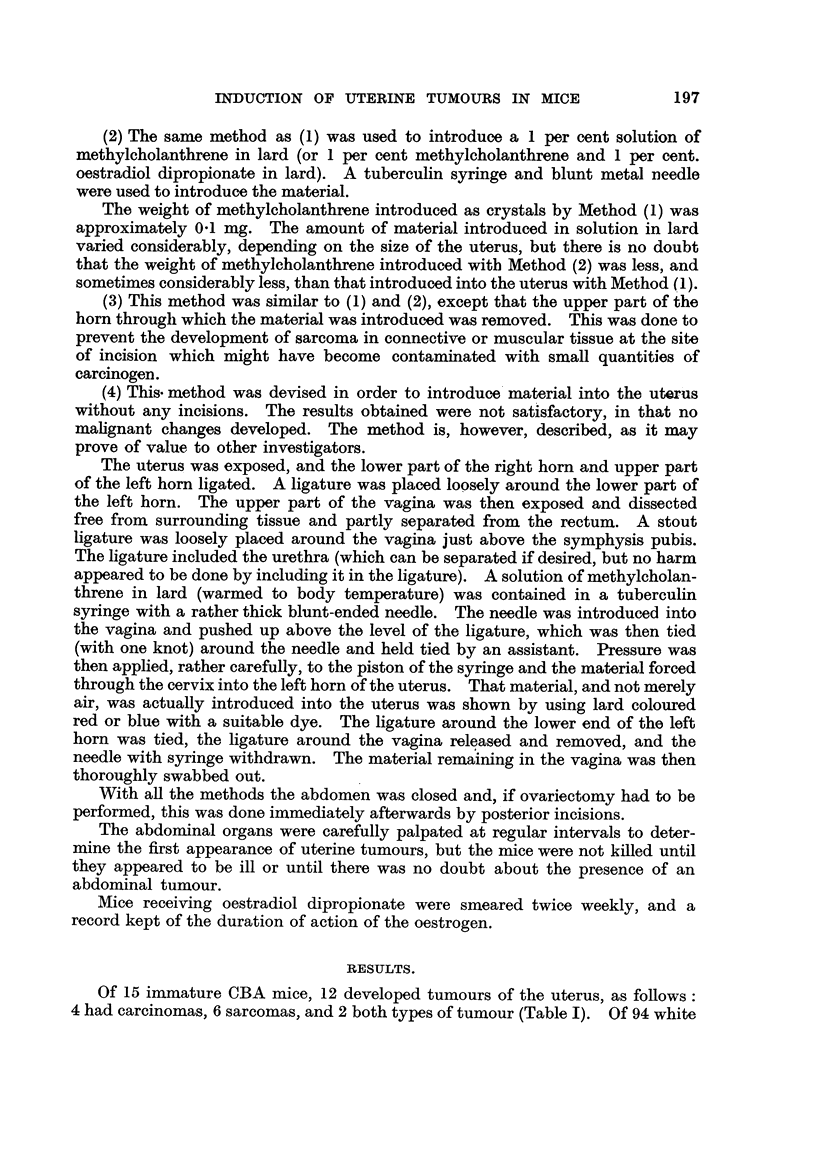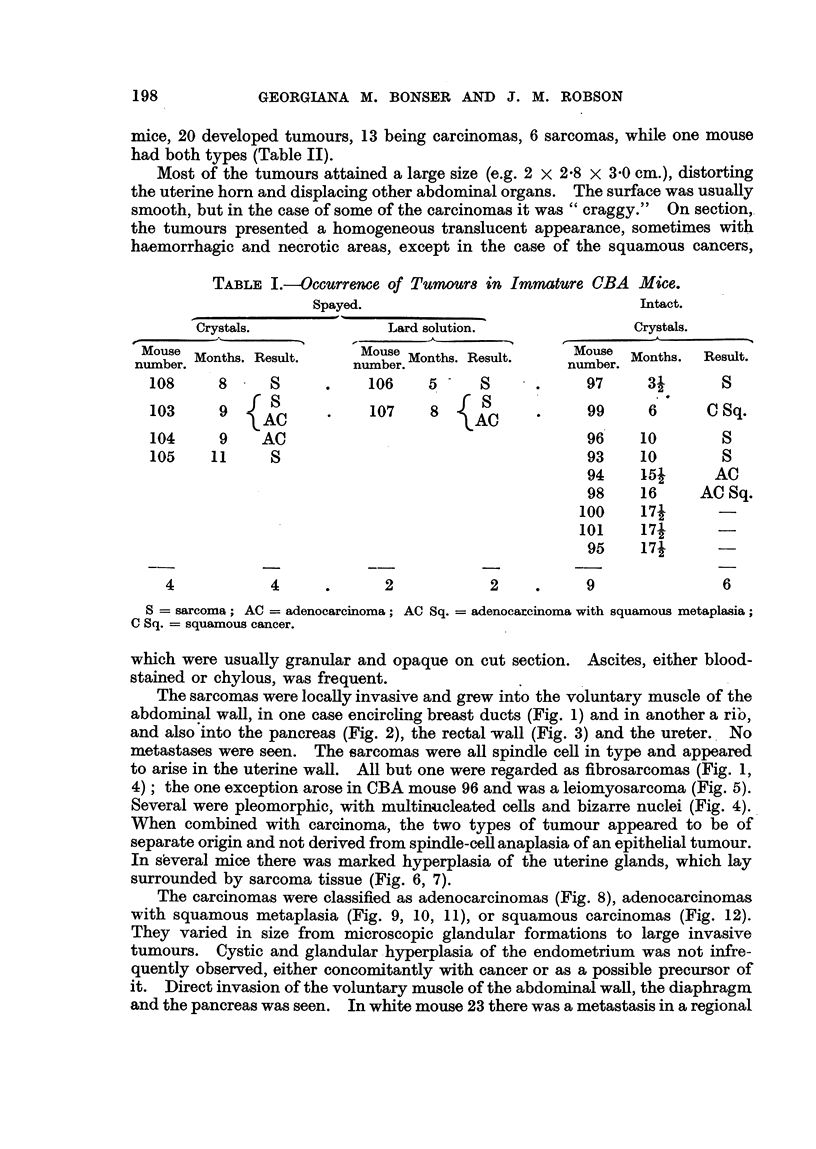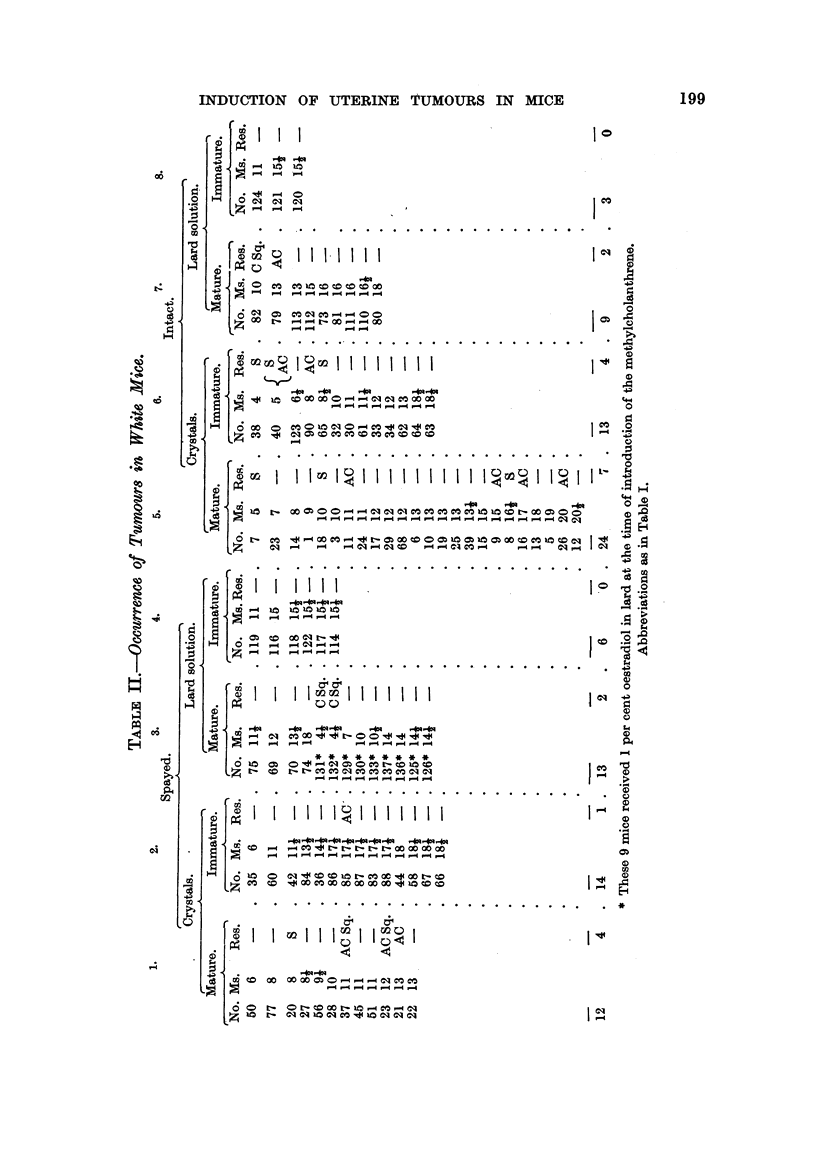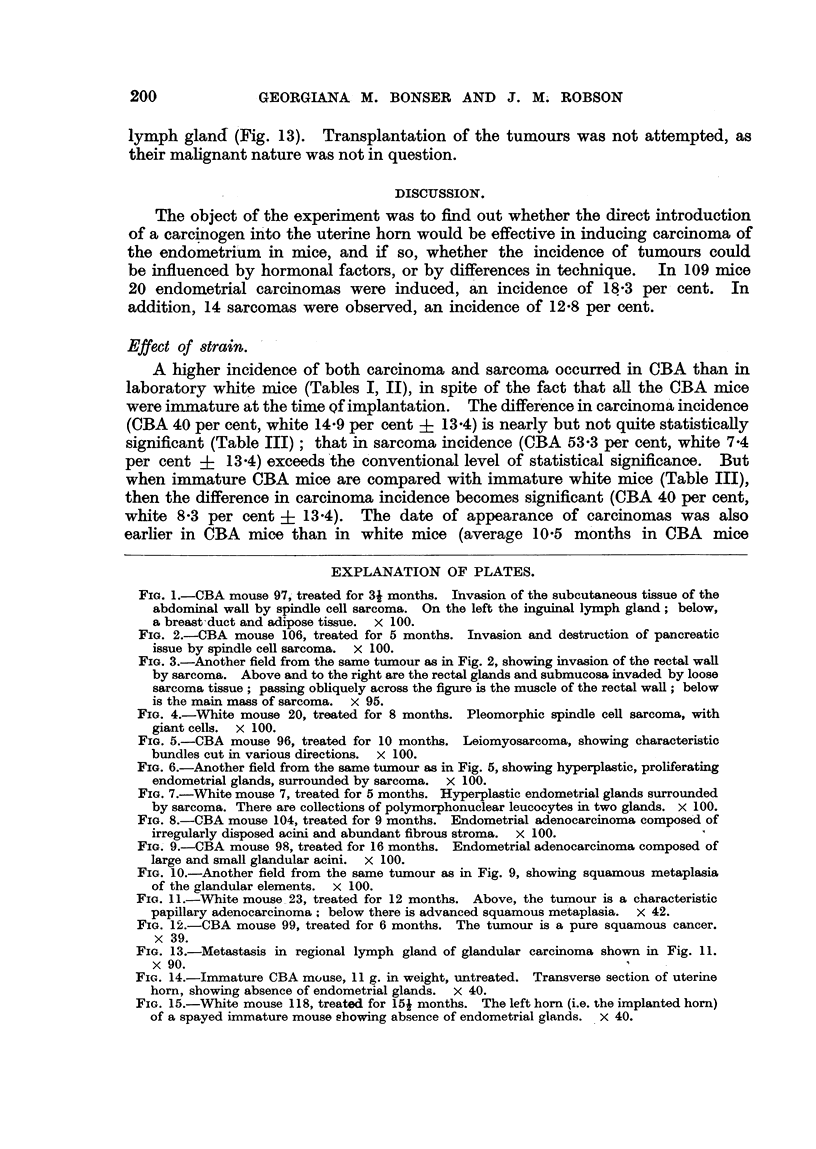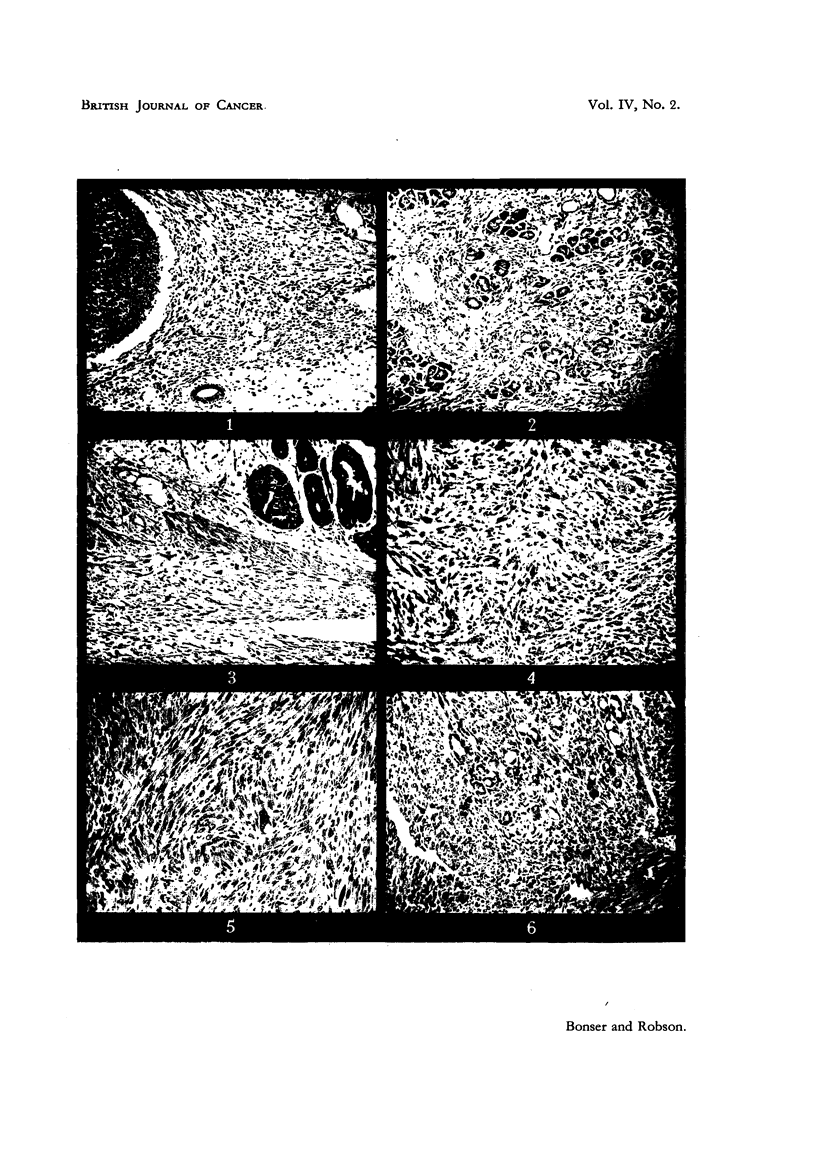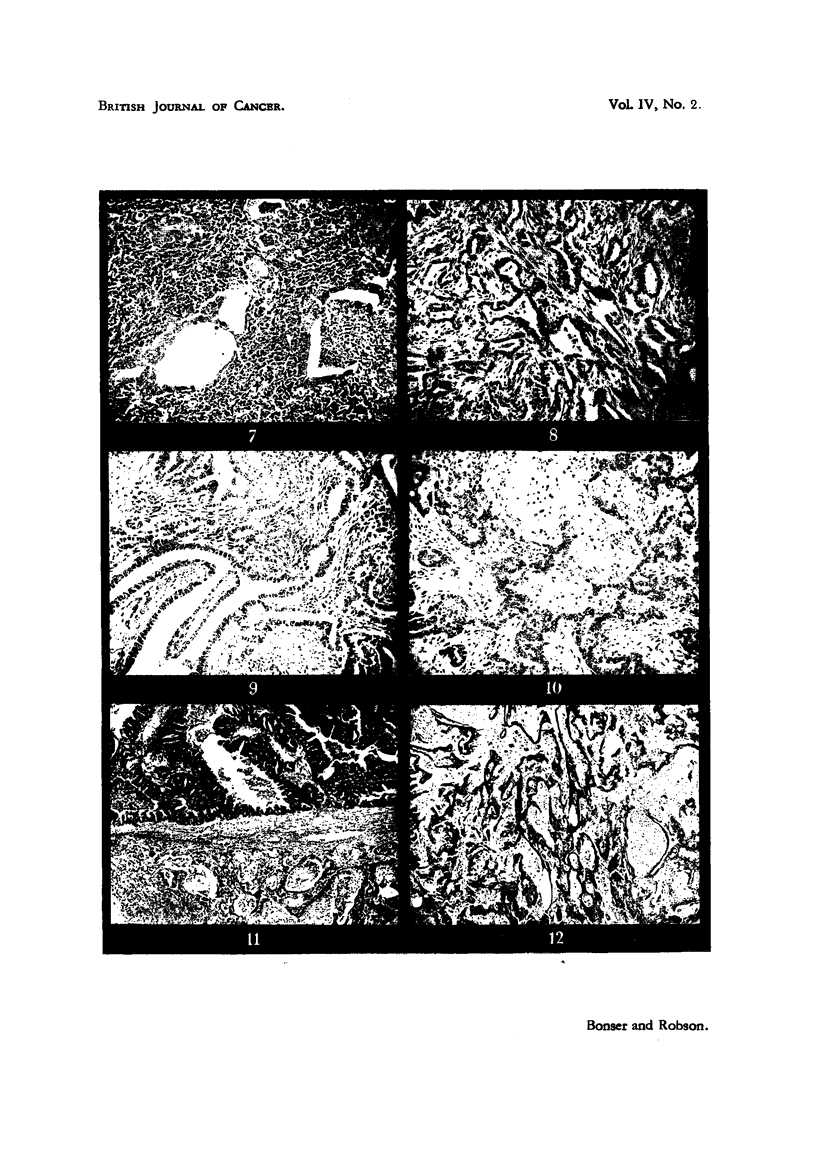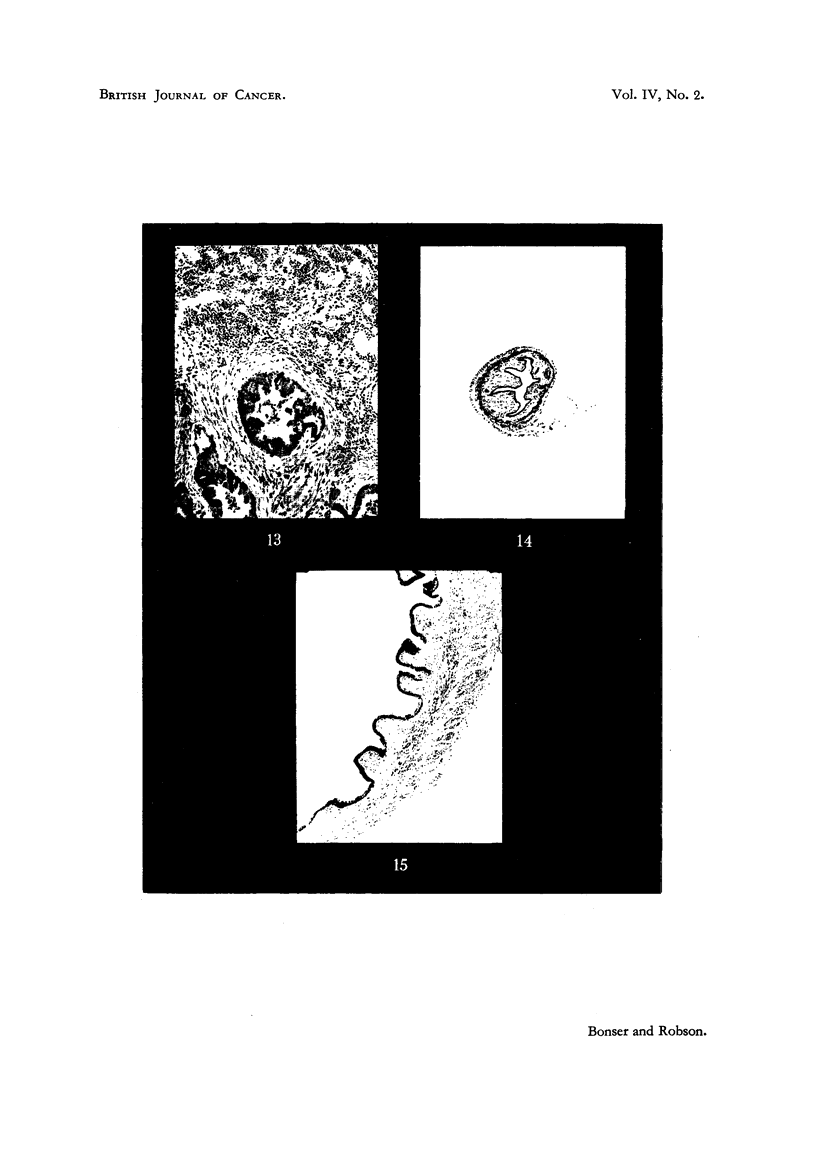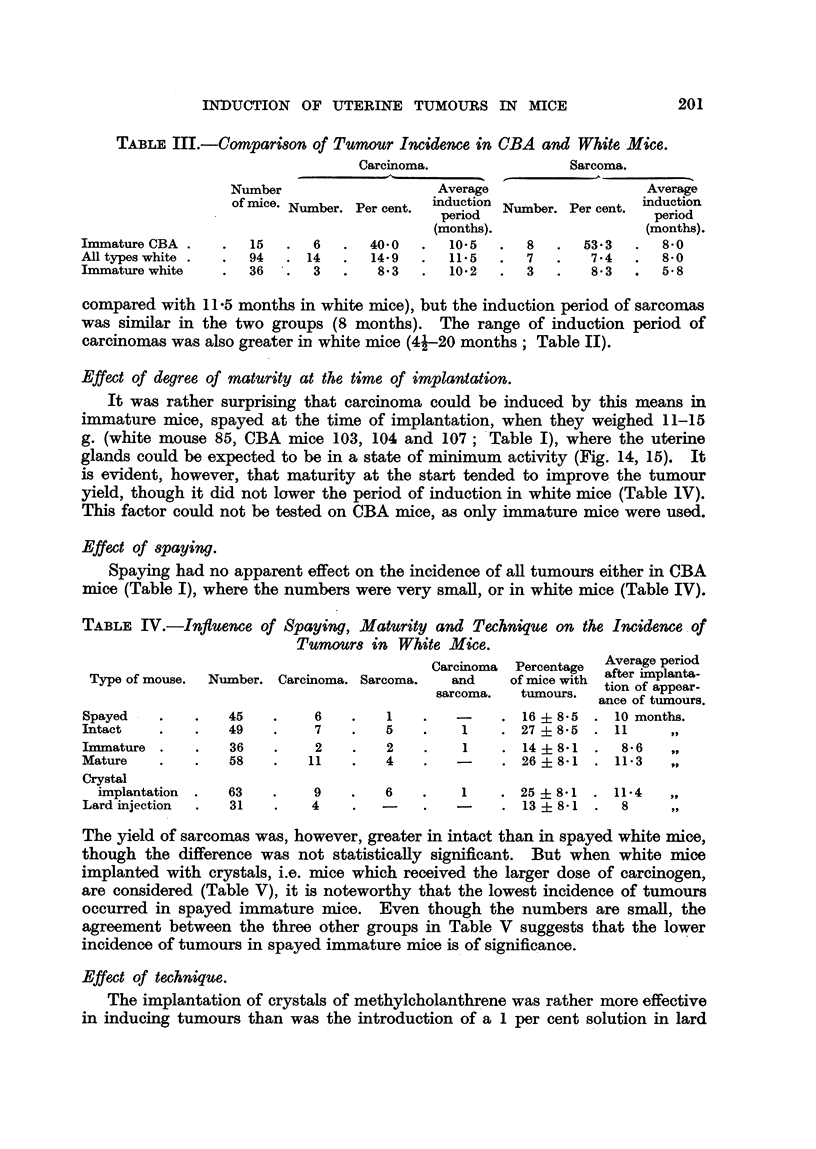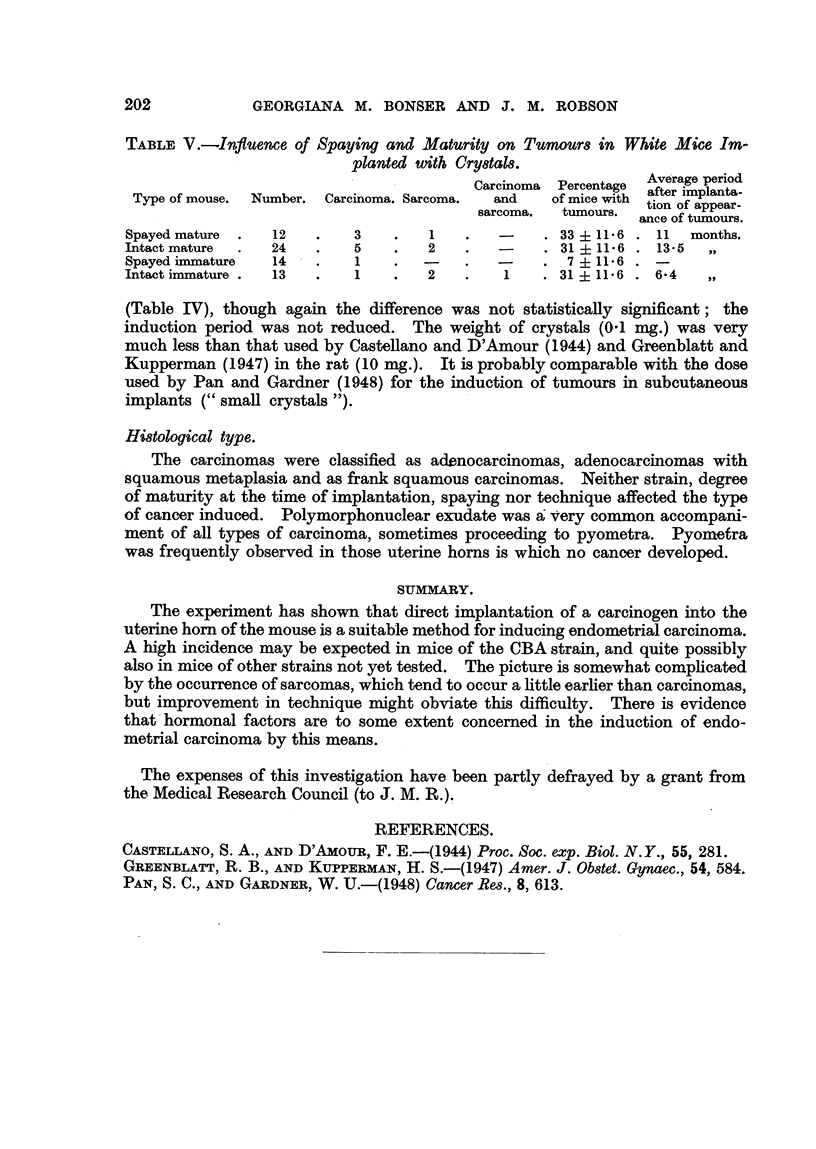# The Induction of Tumours Following the Direct Implantation of 20-Methylcholanthrene into the Uterus of Mice

**DOI:** 10.1038/bjc.1950.19

**Published:** 1950-06

**Authors:** Georgiana M. Bonser, J. M. Robson

## Abstract

**Images:**


### THE INDUCTION OF TUMOURS FOLLOWING THE DIRECT IMPLANTATION OF 20-METHYLCHOLANTHRENE INTO THE UTERUS OF MICE.

GEORGIANA M. BONSER AND J. M. ROBSON.

*From the Department of Experimental Pathology and Cancer Research, University of Leeds, and Department of Pharmacology, Guy's Hospital Medical School, University of London.*

Received for publication March 13, 1950,

IN 1944 Castellano and D'Amour described uterine tumours in rats following the introduction of 10 mg. of methycholanthrene in paraffin wax into the uterine horn. Simultaneous administration of oestrogen improved the yield, whereas progestin (? progesterone) had a slight inhibitory effect. Greenblatt and Kupperman (1947), also using the rat, observed squamous metaplasia of the horn and a doubtful precancerous lesion in the cervix uteri, but no tumours, when a similar dose of methylcholanthrene was introduced. Pan and Gardner (1948) reported the development of numerous carcinomas and sarcomas in mice of several strains, when subcutaneous implants of cervical and uterine epithelium were made together with small crystals of methylcholanthrene.

In the experiments described here uterine tumours were induced by the introduction of methylcholanthrene directly into the uterine horn in two types of mice and the effects of various hormonal factors were studied.

### METHODS.

The experiments were performed on two types of mice : inbred CBA mice, maintained by brother-sister mating in the Department of Cancer Research at Leeds, and white mice obtained from dealers.

Implantations were performed under ether anaesthesia. The following methods were used, and found satisfactory in animals weighing as little as 9 g.:

(1) The abdomen was opened and the viscera gently pushed aside by means of a swab. A fine silk suture was passed around the upper part of the left horn and fastened in such a way that the whole horn became readily accessible. The lower part of the horn was then ligated, and a small incision made with fine scissors into the upper part. In the earlier experiments a " gun," i.e. a fine glass tube with accurately fitting rod acting as piston, was charged with crystalline 20-methylcholanthrene (obtained from Light & Co., Ltd.). The gun was introduced through the incision, a fine silk ligature placed around it a short distance below the incision, and the carcinogen pushed out into the lumen of the horn. As the gun was withdrawn the ligature was tightened, the implant of carcinogen thus remaining between the two ligatures. In later experiments a hypodermic needle, without the shoulder, was used as the "gun," and the stilette used as piston. With this method it is easy to introduce the material even in very small uteri.

(2) The same method as (1) was used to introduce a 1 per cent solution of methylcholanthrene in lard (or 1 per cent methylcholanthrene and 1 per cent. oestradiol dipropionate in lard). A tuberculin syringe and blunt metal needle were used to introduce the material.

The weight of methylcholanthrene introduced as crystals by Method (1) was approximately 0·1 mg. The amount of material introduced in solution in lard varied considerably, depending on the size of the uterus, but there is no doubt that the weight of methylcholanthrene introduced with Method (2) was less, and sometimes considerably less, than that introduced into the uterus with Method (1).

(3) This method was similar to (1) and (2), except that the upper part of the horn through which the material was introduced was removed. This was done to prevent the development of sarcoma in connective or muscular tissue at the site of incision which might have become contaminated with small quantities of carcinogen.

(4) This method was devised in order to introduce material into the uterus without any incisions. The results obtained were not satisfactory, in that no malignant changes developed. The method is, however, described, as it may prove of value to other investigators.

The uterus was exposed, and the lower part of the right horn and upper part of the left horn ligated. A ligature was placed loosely around the lower part of the left horn. The upper part of the vagina was then exposed and dissected free from surrounding tissue and partly separated from the rectum. A stout ligature was loosely placed around the vagina just above the symphysis pubis. The ligature included the urethra (which can be separated if desired, but no harm appeared to be done by including it in the ligature). A solution of methylcholanthrene in lard (warmed to body temperature) was contained in a tuberculin syringe with a rather thick blunt-ended needle. The needle was introduced into the vagina and pushed up above the level of the ligature, which was then tied (with one knot) around the needle and held tied by an assistant. Pressure was then applied, rather carefully, to the piston of the syringe and the material forced through the cervix into the left horn of the uterus. That material, and not merely air, was actually introduced into the uterus was shown by using lard coloured red or blue with a suitable dye. The ligature around the lower end of the left horn was tied, the ligature around the vagina released and removed, and the needle with syringe withdrawn. The material remaining in the vagina was then thoroughly swabbed out.

With all the methods the abdomen was closed and, if ovariectomy had to be performed, this was done immediately afterwards by posterior incisions.

The abdominal organs were carefully palpated at regular intervals to determine the first appearance of uterine tumours, but the mice were not killed until they appeared to be ill or until there was no doubt about the presence of an abdominal tumour.

Mice receiving oestradiol dipropionate were smeared twice weekly, and a record kept of the duration of action of the oestrogen.

### RESULTS.

Of 15 immature CBA mice, 12 developed tumours of the uterus, as follows: 4 had carcinomas, 6 sarcomas, and 2 both types of tumour (Table I). Of 94 white mice, 20 developed tumours, 13 being carcinomas, 6 sarcomas, while one mouse had both types (Table II).

Most of the tumours attained a large size (e.g. 2 × 2·8 × 3·0 cm.), distorting the uterine horn and displacing other abdominal organs. The surface was usually smooth, but in the case of some of the carcinomas it was " craggy." On section, the tumours presented a homogeneous translucent appearance, sometimes with haemorrhagic and necrotic areas, except in the case of the squamous cancers,

TABLE I.—*Occurrence of Tumours in Immature CBA Mice.*

| Spayed. | | | | | | Intact. | | |
|---|---|---|---|---|---|---|---|---|
| Crystals. | | | Lard solution. | | | Crystals. | | |
| Mouse number. | Months. | Result. | Mouse number. | Months. | Result. | Mouse number. | Months. | Result. |
| 108 | 8 | S | 106 | 5 | S | 97 | 3½ | S |
| 103 | 9 | S, AC | 107 | 8 | S, AC | 99 | 6 | C Sq. |
| 104 | 9 | AC | | | | 96 | 10 | S |
| 105 | 11 | S | | | | 93 | 10 | S |
| | | | | | | 94 | 15½ | AC |
| | | | | | | 98 | 16 | AC Sq. |
| | | | | | | 100 | 17½ | — |
| | | | | | | 101 | 17½ | — |
| | | | | | | 95 | 17½ | — |
| 4 | | 4 | 2 | | 2 | 9 | | 6 |

S = sarcoma ; AC = adenocarcinoma ; AC Sq. = adenocarcinoma with squamous metaplasia ; C Sq. = squamous cancer.

which were usually granular and opaque on cut section. Ascites, either blood-stained or chylous, was frequent.

The sarcomas were locally invasive and grew into the voluntary muscle of the abdominal wall, in one case encircling breast ducts (Fig. 1) and in another a rib, and also into the pancreas (Fig. 2), the rectal wall (Fig. 3) and the ureter. No metastases were seen. The sarcomas were all spindle cell in type and appeared to arise in the uterine wall. All but one were regarded as fibrosarcomas (Fig. 1, 4) ; the one exception arose in CBA mouse 96 and was a leiomyosarcoma (Fig. 5). Several were pleomorphic, with multinucleated cells and bizarre nuclei (Fig. 4). When combined with carcinoma, the two types of tumour appeared to be of separate origin and not derived from spindle-cell anaplasia of an epithelial tumour. In several mice there was marked hyperplasia of the uterine glands, which lay surrounded by sarcoma tissue (Fig. 6, 7).

The carcinomas were classified as adenocarcinomas (Fig. 8), adenocarcinomas with squamous metaplasia (Fig. 9, 10, 11), or squamous carcinomas (Fig. 12). They varied in size from microscopic glandular formations to large invasive tumours. Cystic and glandular hyperplasia of the endometrium was not infrequently observed, either concomitantly with cancer or as a possible precursor of it. Direct invasion of the voluntary muscle of the abdominal wall, the diaphragm and the pancreas was seen. In white mouse 23 there was a metastasis in a regional

**TABLE II.—*Occurrence of Tumours in White Mice.***

| Spayed. | | | | | | | | | | | | Intact. | | | | | | | | | | | |
|---|---|---|---|---|---|---|---|---|---|---|---|---|---|---|---|---|---|---|---|---|---|---|---|
| 1. | | | 2. | | | 3. | | | 4. | | | 5. | | | 6. | | | 7. | | | 8. | | |
| Crystals. | | | | | | Lard solution. | | | | | | Crystals. | | | | | | Lard solution. | | | | | |
| Mature. | | | Immature. | | | Mature. | | | Immature. | | | Mature. | | | Immature. | | | Mature. | | | Immature. | | |
| No. | Ms. | Res. | No. | Ms. | Res. | No. | Ms. | Res. | No. | Ms. | Res. | No. | Ms. | Res. | No. | Ms. | Res. | No. | Ms. | Res. | No. | Ms. | Res. |
| 50 | 6 | — | 35 | 6 | — | 75 | 11½ | — | 119 | 11 | — | 7 | 5 | S | 38 | 4 | S | 82 | 10 | C Sq. | 124 | 11 | — |
| 77 | 8 | — | 60 | 11 | — | 69 | 12 | — | 116 | 15 | — | 23 | 7 | — | 40 | 5 | S, AC | 79 | 13 | AC | 121 | 15½ | — |
| 20 | 8 | S | 42 | 11½ | — | 70 | 13½ | — | 118 | 15½ | — | 14 | 8 | — | 123 | 6½ | — | 113 | 13 | — | 120 | 15½ | — |
| 27 | 8½ | — | 84 | 13½ | — | 74 | 18 | — | 122 | 15½ | — | 1 | 9 | — | 90 | 8 | AC | 112 | 15 | — | | | |
| 56 | 9½ | — | 36 | 14½ | — | 131* | 4½ | C Sq. | 117 | 15½ | — | 18 | 10 | S | 65 | 8½ | S | 73 | 16 | — | | | |
| 28 | 10 | — | 86 | 17½ | — | 132* | 4½ | C Sq. | 114 | 15½ | — | 3 | 10 | — | 32 | 10 | — | 81 | 16 | — | | | |
| 37 | 11 | AC Sq. | 85 | 17½ | AC | 129* | 7 | — | | | | 11 | 11 | AC | 30 | 11 | — | 111 | 16 | — | | | |
| 45 | 11 | — | 87 | 17½ | — | 130* | 10 | — | | | | 24 | 11 | — | 61 | 11½ | — | 110 | 16½ | — | | | |
| 51 | 11 | — | 83 | 17½ | — | 133* | 10½ | — | | | | 17 | 12 | — | 33 | 12 | — | 80 | 18 | — | | | |
| 23 | 12 | AC Sq. | 88 | 17½ | — | 137* | 14 | — | | | | 29 | 12 | — | 34 | 12 | — | | | | | | |
| 21 | 13 | AC | 44 | 18 | — | 136* | 14 | — | | | | 68 | 12 | — | 62 | 13 | — | | | | | | |
| 22 | 13 | — | 58 | 18½ | — | 125* | 14½ | — | | | | 6 | 13 | — | 64 | 18½ | — | | | | | | |
| | | | 67 | 18½ | — | 126* | 14½ | — | | | | 10 | 13 | — | 63 | 18½ | — | | | | | | |
| | | | 66 | 18½ | — | | | | | | | 19 | 13 | — | | | | | | | | | |
| | | | | | | | | | | | | 25 | 13 | — | | | | | | | | | |
| | | | | | | | | | | | | 39 | 13½ | — | | | | | | | | | |
| | | | | | | | | | | | | 15 | 15 | — | | | | | | | | | |
| | | | | | | | | | | | | 9 | 15 | AC | | | | | | | | | |
| | | | | | | | | | | | | 8 | 16½ | S | | | | | | | | | |
| | | | | | | | | | | | | 16 | 17 | AC | | | | | | | | | |
| | | | | | | | | | | | | 13 | 18 | — | | | | | | | | | |
| | | | | | | | | | | | | 5 | 19 | — | | | | | | | | | |
| | | | | | | | | | | | | 26 | 20 | AC | | | | | | | | | |
| | | | | | | | | | | | | 12 | 20½ | — | | | | | | | | | |
| 12 | | 4 | 14 | | 1 | 13 | | 2 | 6 | | 0 | 24 | | 7 | 13 | | 4 | 9 | | 2 | 3 | | 0 |

* These 9 mice received 1 per cent oestradiol in lard at the time of introduction of the methylcholanthrene.

Abbreviations as in Table I.

lymph gland (Fig. 13). Transplantation of the tumours was not attempted, as their malignant nature was not in question.

### DISCUSSION.

The object of the experiment was to find out whether the direct introduction of a carcinogen into the uterine horn would be effective in inducing carcinoma of the endometrium in mice, and if so, whether the incidence of tumours could be influenced by hormonal factors, or by differences in technique. In 109 mice 20 endometrial carcinomas were induced, an incidence of 18·3 per cent. In addition, 14 sarcomas were observed, an incidence of 12·8 per cent.

*Effect of strain.*

A higher incidence of both carcinoma and sarcoma occurred in CBA than in laboratory white mice (Tables I, II), in spite of the fact that all the CBA mice were immature at the time of implantation. The difference in carcinoma incidence (CBA 40 per cent, white 14·9 per cent $\pm$ 13·4) is nearly but not quite statistically significant (Table III) ; that in sarcoma incidence (CBA 53·3 per cent, white 7·4 per cent $\pm$ 13·4) exceeds the conventional level of statistical significance. But when immature CBA mice are compared with immature white mice (Table III), then the difference in carcinoma incidence becomes significant (CBA 40 per cent, white 8·3 per cent $\pm$ 13·4). The date of appearance of carcinomas was also earlier in CBA mice than in white mice (average 10·5 months in CBA mice

---

### EXPLANATION OF PLATES.

FIG. 1.—CBA mouse 97, treated for 3½ months. Invasion of the subcutaneous tissue of the abdominal wall by spindle cell sarcoma. On the left the inguinal lymph gland ; below, a breast duct and adipose tissue. × 100.

FIG. 2.—CBA mouse 106, treated for 5 months. Invasion and destruction of pancreatic issue by spindle cell sarcoma. × 100.

FIG. 3.—Another field from the same tumour as in Fig. 2, showing invasion of the rectal wall by sarcoma. Above and to the right are the rectal glands and submucosa invaded by loose sarcoma tissue ; passing obliquely across the figure is the muscle of the rectal wall ; below is the main mass of sarcoma. × 95.

FIG. 4.—White mouse 20, treated for 8 months. Pleomorphic spindle cell sarcoma, with giant cells. × 100.

FIG. 5.—CBA mouse 96, treated for 10 months. Leiomyosarcoma, showing characteristic bundles cut in various directions. × 100.

FIG. 6.—Another field from the same tumour as in Fig. 5, showing hyperplastic, proliferating endometrial glands, surrounded by sarcoma. × 100.

FIG. 7.—White mouse 7, treated for 5 months. Hyperplastic endometrial glands surrounded by sarcoma. There are collections of polymorphonuclear leucocytes in two glands. × 100.

FIG. 8.—CBA mouse 104, treated for 9 months. Endometrial adenocarcinoma composed of irregularly disposed acini and abundant fibrous stroma. × 100.

FIG. 9.—CBA mouse 98, treated for 16 months. Endometrial adenocarcinoma composed of large and small glandular acini. × 100.

FIG. 10.—Another field from the same tumour as in Fig. 9, showing squamous metaplasia of the glandular elements. × 100.

FIG. 11.—White mouse 23, treated for 12 months. Above, the tumour is a characteristic papillary adenocarcinoma ; below there is advanced squamous metaplasia. × 42.

FIG. 12.—CBA mouse 99, treated for 6 months. The tumour is a pure squamous cancer. × 39.

FIG. 13.—Metastasis in regional lymph gland of glandular carcinoma shown in Fig. 11. × 90.

FIG. 14.—Immature CBA mouse, 11 g. in weight, untreated. Transverse section of uterine horn, showing absence of endometrial glands. × 40.

FIG. 15.—White mouse 118, treated for 15½ months. The left horn (i.e. the implanted horn) of a spayed immature mouse showing absence of endometrial glands. × 40.

1
2
3
4
5
6

7
8
9
10
11
12

13

14

15

Bonser and Robson.

TABLE III.—*Comparison of Tumour Incidence in CBA and White Mice.*

| | Number of mice. | Carcinoma. Number. | Carcinoma. Per cent. | Carcinoma. Average induction period (months). | Sarcoma. Number. | Sarcoma. Per cent. | Sarcoma. Average induction period (months). |
|---|---|---|---|---|---|---|---|
| Immature CBA | 15 | 6 | 40·0 | 10·5 | 8 | 53·3 | 8·0 |
| All types white | 94 | 14 | 14·9 | 11·5 | 7 | 7·4 | 8·0 |
| Immature white | 36 | 3 | 8·3 | 10·2 | 3 | 8·3 | 5·8 |

compared with 11·5 months in white mice), but the induction period of sarcomas was similar in the two groups (8 months). The range of induction period of carcinomas was also greater in white mice (4½–20 months ; Table II).

*Effect of degree of maturity at the time of implantation.*

It was rather surprising that carcinoma could be induced by this means in immature mice, spayed at the time of implantation, when they weighed 11–15 g. (white mouse 85, CBA mice 103, 104 and 107 ; Table I), where the uterine glands could be expected to be in a state of minimum activity (Fig. 14, 15). It is evident, however, that maturity at the start tended to improve the tumour yield, though it did not lower the period of induction in white mice (Table IV). This factor could not be tested on CBA mice, as only immature mice were used.

*Effect of spaying.*

Spaying had no apparent effect on the incidence of all tumours either in CBA mice (Table I), where the numbers were very small, or in white mice (Table IV).

TABLE IV.—*Influence of Spaying, Maturity and Technique on the Incidence of Tumours in White Mice.*

| Type of mouse. | Number. | Carcinoma. | Sarcoma. | Carcinoma and sarcoma. | Percentage of mice with tumours. | Average period after implantation of appearance of tumours. |
|---|---|---|---|---|---|---|
| Spayed | 45 | 6 | 1 | — | $16 \pm 8 \cdot 5$ | 10 months. |
| Intact | 49 | 7 | 5 | 1 | $27 \pm 8 \cdot 5$ | 11 " |
| Immature | 36 | 2 | 2 | 1 | $14 \pm 8 \cdot 1$ | 8·6 " |
| Mature | 58 | 11 | 4 | — | $26 \pm 8 \cdot 1$ | 11·3 " |
| Crystal implantation | 63 | 9 | 6 | 1 | $25 \pm 8 \cdot 1$ | 11·4 " |
| Lard injection | 31 | 4 | — | — | $13 \pm 8 \cdot 1$ | 8 " |

The yield of sarcomas was, however, greater in intact than in spayed white mice, though the difference was not statistically significant. But when white mice implanted with crystals, i.e. mice which received the larger dose of carcinogen, are considered (Table V), it is noteworthy that the lowest incidence of tumours occurred in spayed immature mice. Even though the numbers are small, the agreement between the three other groups in Table V suggests that the lower incidence of tumours in spayed immature mice is of significance.

*Effect of technique.*

The implantation of crystals of methylcholanthrene was rather more effective in inducing tumours than was the introduction of a 1 per cent solution in lard

TABLE V.—*Influence of Spaying and Maturity on Tumours in White Mice Implanted with Crystals.*

| Type of mouse. | Number. | Carcinoma. | Sarcoma. | Carcinoma and sarcoma. | Percentage of mice with tumours. | Average period after implantation of appearance of tumours. |
|---|---|---|---|---|---|---|
| Spayed mature | 12 | 3 | 1 | — | 33 ± 11·6 | 11 months. |
| Intact mature | 24 | 5 | 2 | — | 31 ± 11·6 | 13·5 „ |
| Spayed immature | 14 | 1 | — | — | 7 ± 11·6 | — |
| Intact immature | 13 | 1 | 2 | 1 | 31 ± 11·6 | 6·4 „ |

(Table IV), though again the difference was not statistically significant; the induction period was not reduced. The weight of crystals (0·1 mg.) was very much less than that used by Castellano and D'Amour (1944) and Greenblatt and Kupperman (1947) in the rat (10 mg.). It is probably comparable with the dose used by Pan and Gardner (1948) for the induction of tumours in subcutaneous implants (" small crystals ").

*Histological type.*

The carcinomas were classified as adenocarcinomas, adenocarcinomas with squamous metaplasia and as frank squamous carcinomas. Neither strain, degree of maturity at the time of implantation, spaying nor technique affected the type of cancer induced. Polymorphonuclear exudate was a very common accompaniment of all types of carcinoma, sometimes proceeding to pyometra. Pyometra was frequently observed in those uterine horns is which no cancer developed.

SUMMARY.

The experiment has shown that direct implantation of a carcinogen into the uterine horn of the mouse is a suitable method for inducing endometrial carcinoma. A high incidence may be expected in mice of the CBA strain, and quite possibly also in mice of other strains not yet tested. The picture is somewhat complicated by the occurrence of sarcomas, which tend to occur a little earlier than carcinomas, but improvement in technique might obviate this difficulty. There is evidence that hormonal factors are to some extent concerned in the induction of endometrial carcinoma by this means.

The expenses of this investigation have been partly defrayed by a grant from the Medical Research Council (to J. M. R.).

REFERENCES.

CASTELLANO, S. A., AND D'AMOUR, F. E.—(1944) *Proc. Soc. exp. Biol. N.Y.*, **55**, 281.

GREENBLATT, R. B., AND KUPPERMAN, H. S.—(1947) *Amer. J. Obstet. Gynaec.*, **54**, 584.

PAN, S. C., AND GARDNER, W. U.—(1948) *Cancer Res.*, **8**, 613.